# ﻿An updated checklist of Collembola in Taiwan, with DNA barcoding of *Papirioidesjacobsoni* Folsom, 1924 (Symphypleona, Dicyrtomidae)

**DOI:** 10.3897/zookeys.1123.90202

**Published:** 2022-10-04

**Authors:** Hsin-Ju Cheng, Frans Janssens, Chih-Han Chang

**Affiliations:** 1 Institute of Ecology and Evolutionary Biology, National Taiwan University, Taipei, Taiwan; 2 Evolutionary Ecology, Department of Biology, University of Antwerp, Groenenborgerlaan 171, 2020 Antwerpen, Belgium; 3 Department of Life Science, National Taiwan University, Taipei, Taiwan

**Keywords:** Biodiversity, Entomobryomorpha, Hexapoda, Poduromorpha, springtail

## Abstract

From urban green space to pristine forest, Collembola is one of the most numerous and species-rich members of the soil fauna around the world. However, due to lack of taxonomic expertise and research, its diversity is poorly understood, especially in tropical and subtropical regions. Collembola biodiversity studies in Taiwan have not seen much progress since 1981, when Hsin Chi reviewed 26 species belonging to 20 genera and eight families. Additionally, reports of new records in Taiwan in the last 40 years are scattered amongst several publications and not easily accessible to most end-users. Thus, a concise summary of related research is urgently needed. In this study, we updated the checklist of Collembola in Taiwan, based on published papers as well as images recorded in 2020–2022. We concluded that 58 species of Collembola belonging to 31 genera and 12 families have been reported in Taiwan, including 13 newly-recorded species. This species richness marks a 123% increase from the 1981 review. The results have been made publicly available in the Catalog of Life in Taiwan database and the images recorded have been used to update species information in collembola.org. We also characterised morphological and genetic variations in the globular springtail species *Papirioidesjacobsoni* Folsom, 1924 using DNA barcodes and highlighted potential research directions.

## ﻿Introduction

Springtails are microarthropods in the class Collembola (Arthropoda: Hexapoda). They are commonly found in leaf litter and soil and on the surface of plants, fungal sporocarp, decaying wood and rocks. They are one of the most abundant animals in the litter-soil habitat, with a density of up to 40,000 individuals per square metre in the soil in temperate grasslands or forests (Orgiazzi et al. 2016). Their morphologies are characterised by ventral tube/collophore on the first abdominal segment, which helps anchor themselves to the surface, as well as furca/furcula, the structure allowing them to jump. In some taxonomic groups, this latter structure is reduced and, therefore, species in these groups lost the ability to jump. Globally, about 9,000 species of Collembola have been described so far and the estimated number of species is about 50,000 to 65,000 (Bellinger et al. 1996–2022). Most of our knowledge about this diversity comes from studies conducted in the temperate region, whereas the subtropical region has received little attention (Potapov et al. 2020).

Taiwan is an East Asian Island located between Japan and the Philippines. It has a land area of about 32,260 km^2^ and is divided by the Tropic of Cancer into a humid subtropical climate in the north and a tropical monsoon climate in the south, with a mean annual precipitation of approximately 2,600 mm, mostly in the form of rainfall. The terrain on the Island was shaped by the collision between the Eurasian Plate and the Philippine Sea Plate in the last five million years (Huang et al. 1997, 2000). Geographically, it is divided into the flat to gently rolling plains in the west and the rugged, forest-covered mountains in the eastern two-thirds of the Island, with over 100 mountain peaks exceeding 3,000 m in elevation. Some of these summits were covered by glaciers during the last glaciation (Ono et al. 2005) and are still regularly receiving snow and short periods of ice cover during winter nowadays. The complex terrain, climate and geological history of Taiwan, presumably, provide ample opportunity for the diversification of Collembola, as well as varying vegetation and habitats for these organisms to thrive.

Following “An Index to the Collembola” for scientific names (Salmon 1964), the first and by far the only review of Collembola in Taiwan was a Chinese-written article by Hsin Chi in 1981, which listed 26 species belonging to 20 genera and eight families (Chi 1981). As Chi noted, studies of Collembola in Taiwan during the early years were mainly conducted by Japanese researchers. The first publication was by J.R. Denis (1929), which reported three species collected from Taipei by F. Silvestri. After that, Japanese taxonomists R. Yosii and H. Uchida reported several species of Collembola in Japan and neighbouring countries and up to 37 species from Taiwan were included (Yosii 1940, 1963, 1965, 1977; Uchida 1943, 1955, 1956, 1957a, 1957b, 1958a, 1958b, 1959a, 1959b, 1960). As some of the records were later considered synonyms, the total number of species reported during this period was higher than that in the checklist compiled by Chi (1981). In addition, some Taiwanese species were occasionally recorded in entomological literature (Shiraki 1932, 1954; Asahina et al. 1965) and an article about sugar cane pests (Takano and Yanagihara 1939).

In addition to Chi’s (1981) comprehensive checklist, another 22 species have been reported in Taiwan by researchers from China, Korea and Japan. Lee and Park (1989) reported 11 species and seven genera in family Entomobryidae, including four new species and three new records. A year later, Lee and Kim (1990) reported five new species and two new records in family Neanuridae. In 2010, a subspecies of *Homidia* (Entomobryidae) was re-described and elevated to species level (Shi et al. 2010). Moreover, several new records were sporadically reported (Yosii 1966, 1982; Zhao et al. 1997). In contrast, studies conducted by Taiwanese researchers were mainly about pest control or survey of ground or soil arthropods, which only recorded the total number of individuals of Collembola without any detailed taxonomic information (Chen et al. 2020).

Taken together, our knowledge on the diversity of Taiwanese collembolan fauna has changed considerably in the last 40 years since Chi’s comprehensive review, including changes in scientific names and synonyms. In this study, we updated the checklist of Collembola in Taiwan, based on published papers as well as images we recorded in 2020–2022. During our field sampling, we noticed apparent variations in the colour pattern of the species *Papirioidesjacobsoni* Folsom, 1924, calling into question whether the different colour morphs are, indeed, the same species. Thus, we hypothesised that these colour morphs represent two different species and conducted DNA barcode analysis to test this hypothesis.

## ﻿Materials and methods

The revised checklist is based on both published studies and newly-collected samples. Most of the sampling sites are hiking trails in forests and urban areas in northern Taiwan, with only a few samples from eastern and central Taiwan. Collembola were collected using one of the two methods; (1) Litter and surface soil were collected and then transported to the laboratory within 24 hours. Collembola were extracted from litter and soil using a Berlese-Tullgren funnel for about 5–7 days. Specimens were extracted into either a jar containing 85% ethanol or a container filled with the mixture of Plaster of Paris and fine powder of activated charcoal (Plaster of Paris: activated carbon: water = 9:1:11.25); (2) For specimens that were directly spotted in the field, an aspirator was used to collect them. The collected specimens were either kept alive for as long as possible in a container filled with the mixture of Plaster of Paris and activated charcoal or stored in 85% ethanol at 4 °C for future molecular study.

Live and ethanol-preserved specimens were examined under a Nikon SMZ800N stereomicroscope, equipped with a plan Apo 1× objective lens to reduce chromatic aberration and a TOUPCAM E3ISPM12300KPA digital camera for photography. Species identification is based on Bretfeld (1999), Potapov (2001) and Jordana (2012). For families, scientific names and synonyms, we followed the Checklist of the Collembola of the World maintained by Bellinger et al. (1996–2022) and hosted in collembola.org. In most cases, junior synonyms were listed when they were related to previous records of Taiwanese Collembola. Whenever available, additional information about locations and habitats of a species was detailed in the Remarks. Species marked with an asterisk (*) are new records identified based on photographs of live specimens collected by the Taiwanese authors.

For molecular analysis, genomic DNA was extracted from whole specimens of *Papirioidesjacobsoni* using the QIAamp DNA Micro Kit (Qiagen, Hilden, Germany) following the manufacturer’s instruction. Before extraction, 1μl of carrier RNA was add into buffer AL. The extracted DNA was eluted in 50 μl elution buffer and stored at –20 °C. Polymerase chain reaction (PCR) for the mitochondrial cytochrome *c* oxidase subunit 1 gene (COI), the DNA barcode for animals, was conducted using the primers LCO1490 and HCO2198 (Folmer et al. 1994) in a 20-μl volume containing 0.2 mM dNTP, 0.5 μM of each primer, 1.5 mM MgCl_2_, 1.28 μg/μl BSA and 1 U Taq polymerase. Amplification was carried out with a preheat at 94 °C for 1 min, followed by 5 cycles of 94 °C for 30 sec, 45 °C for 30 sec and 72 °C for 50 sec and then by 35 cycles of 94 °C for 30 sec, 51 °C for 30 sec and 72 °C for 50 sec, with a final extension at 72 °C for 10 min. PCR products were checked using 1.5% agarose gel electrophoresis and sequenced by Genomics (Taipei, Taiwan) using an ABI 3730X Genetic Analyzer (Applied Biosystems, CA, USA). DNA sequences were assembled in Geneious (Dotmatics, MA, USA), double-checked by eye and deposited in GenBank under accession numbers ON602032–ON602038.

For DNA barcode analysis, COI sequences of *Dicyrtominaornata* (Nicolet, 1842), *Ptenothrixmaculosa* (Schött, 1891) and *Ptenothrixhuangshanensis* Chen & Christiansen, 1996 were retrieved from GenBank (accession numbers KT808331, KU874836 and MK423965, respectively) and used as outgroups. The acquired sequences were aligned using ClustalX 2.0 (Larkin et al. 2007). A neighbour-joining analysis was conducted using Kimura’s two-parameter model (Kimura 1980) in MEGA X (Kumar et al. 2018), with 1,000 bootstrap pseudo-replicates to evaluate the robustness of clades.

## ﻿Results

### ﻿Checklist and classification

**Class Collembola Lubbock, 1870**

**Order Poduromorpha Börner, 1913**

#### Family Hypogastruridae Börner, 1906

**1. *Ceratophysellaarmata* (Nicolet, 1842)**

*Poduraarmata*Nicolet 1842.

*Achorutesarmatus*: Oudemans 1890, Yosii 1940.

*Hypogastruraarmata*: Chi 1981.

**Remarks.** Mt. Taiping, Datong Township, Yilan County (Yosii 1940).

**2. *Ceratophysellacommunis* (Folsom, 1898)**

Fig. 1A

**Figure 1.**
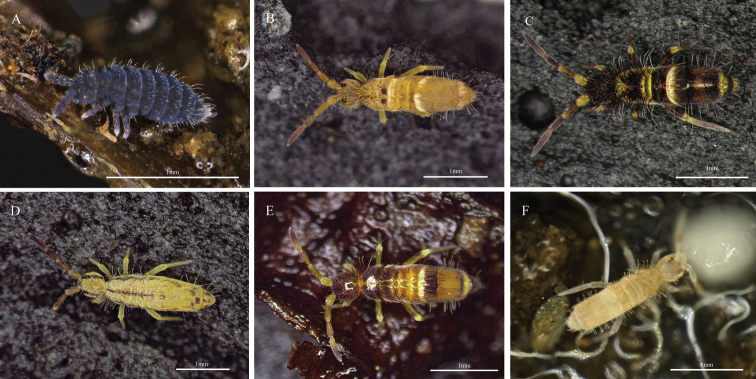
Photos of Collembola in Taiwan **A***Ceratophysellacommunis* (Folsom, 1898) **B***Homidialinhaiensis* Shi, Pan & Qi, 2009 **C***Homidianigrocephala* Uchida, 1943 **D***Homidiasocia* Denis, 1929 **E***Homidiataibaiensis* Yuan & Pan, 2013 **F***Sinellacurviseta* Brook, 1882.

*Achorutescommunis* Folsom, 1898.

*Achorutescommunis*: Yosii 1940.

*Hypogastruracommunis*: Uchida 1956.

*Neogastruracommunis*: Uchida 1965.

*Hypogastruraarmatacommunis*: Chi 1981.

**Remarks.** Taipei (Denis 1929). Collected in Houtong, New Taipei City (25°5'14.62"N, 121°49'38.95"E) on 22 November 2021.

#### Family Neanuridae Börner, 1901

**3. *Crossodonthinaalatoserrata* Yosii, 1965**

*Imparituberculaalatoserrata*: Chi 1981.

**Remarks.** Taipei (Yosii 1965).

**4. *Crossodonthinaformosana* Yosii, 1965**

*Imparituberculaformosana*: Chi 1981.

**Remarks.** Wulai, New Taipei City, from soil and litter of mixed arboreal vegetation (Lee and Kim 1990).

**5. *Crossodonthinamontana* Lee & Kim, 1990**

**Remarks.** Kantaoshan, Nantou County, from soil and litter of mixed arboreal vegetation (Lee and Kim 1990).

**6. *Lobellanana* Lee & Kim, 1990**

**Remarks.** Wushe, Nantou County, from the litter of pine forest (Lee and Kim 1990).

**7. *Neanurakentingensis* Lee & Kim, 1990**

**Remarks.** Kenting Park, Pingtung County, from dry soil under shrubs (Lee and Kim 1990).

**8. *Paleonuraformosana* (Yosii, 1965)**

*Paranuraformosana* Yosii, 1965: Chi 1981.

**Remarks.** Taipei (Yosii 1965).

**9. *Paralobellaperfusa* (Denis, 1934)**

*Lobellaperfusa* Denis, 1934: Lee and Kim 1990.

**Remarks.** Xitou, Nantou County, from bamboo and pine leaf litter and litter and soil of mixed arboreal vegetation and root of herbage (Lee and Kim 1990).

**10. *Pseudachorudinanepalica* Yosii, 1966**

**Remarks.** Xitou, Nantou County, from moss and under stones (Lee and Kim 1990).

**11. *Vitronurarosea* (Gervais, 1842)**

*Anourarosea* Gervais, 1842.

*Achorutesroseus*: Handschin 1929, Uchida 1956.

*Bilobarosea*: Uchida 1965.

*Neanurarosea*: Chi 1981, Lee and Kim 1990.

*Neanuragiselae* Gisin, 1950.

**Remarks.** Locality not specified (Chi 1981; Lee and Kim 1990). Bellinger et al. (1996–2022) noted that “given Yoshii (1995) synonymised *rosea* Gervais with *giselae* Gisin and *mandarina* Yosii, according to ICZN rules of priority, *rosea* Gervais, 1842 takes priority on *giselae* Gisin, 1950 and *mandarina* Yosii, 1954”. Thus, we list the species as *Vitronurarosea*.

**12. *Vitronurapygmaea* (Yosii, 1954)**

*Metanurapygmea* Yosii, 1954.

**Remarks.** Locality not specified (Yosii 1977).

**13. *Vitronurasingaporiensis* (Yosii, 1959)**

*Bilobellasingaporiensis* Yosii, 1959.

**Remarks.** Wulai, New Taipei City (Yosii 1976).

**14. *Vitronuratubercula* Lee & Kim, 1990**

**Remarks.** Wulai, New Taipei City, from soil and litter of mixed arboreal vegetation (Lee and Kim 1990).

**15. *Womersleyaformosana* Lee & Kim, 1990**

**Remarks.** Manchou, Pingtung County, from soil under shrubs (Lee and Kim 1990).

**Family Onychiuridae Lubbock, 1867**

**16. *Formosanonychiurusformosanus* (Denis, 1929)**

*Onychiurusformosanus* Denis, 1929.

*Paronychiurusformosanus*: Chi 1981.

**Remarks.** Taipei (Denis 1929).

#### Family Poduridae Latreille, 1804

**17. *Poduraaquatica* Linnaeus, 1758**

**Remarks.** Cosmopolitan (Usinger 1956). First recorded in Shiraki (1932).

**Order Entomobryomorpha Börner, 1913**

#### Family Entomobryidae Schäffer, 1896

**18. *Dicranocentrusindicus* Bonet, 1930**

**Remarks.**Yosii (1966). Locality unknown.

**19. *Homidiaformosana* Uchida, 1943**

*Homidiasauteriformosana* Uchida, 1943: Chi 1981.

**Remarks.** Meixi, Ren’ai Township, Nantou County (Uchida 1943), from leaf litter of *Liquidambarformosana* (Shi et al. 2010).

*20. *Homidialinhaiensis* Shi, Pan & Qi, 2009

Fig. 1B

**Remarks.** New record. Collected in Xiaokengxi, Wenshan District, Taipei City (24°59'6.06"N, 121°35'5.82"E) on 31 December 2021.

**21. *Homidianigrocephala* Uchida, 1943**

Fig. 1C

**Remarks.** Meixi, Ren’ai Township, Nantou County and Mt. Taiping, Datong Township, Yilan County (Uchida 1943). Collected in Baoshan, Hsinchu County (24°44'32.73"N, 121°03'28.76"E) on 8 October 2020.

**22. *Homidiasauteri* (Börner, 1909)**

Entomobrya (Homidia) sauteri Börner, 1909.

**Remarks.** Locality not specified (Aoki 2015).

**23. *Homidiasocia* Denis, 1929**

Fig. 1D

**Remarks.** Kenting National Park, Pintung County, from soil under shrubs, bamboo leaves, thicket of sugar cane leaves, forest of *Aphanamixis* and lawn (Lee and Park 1989). Collected in Xindian, New Taipei City (24°58'17.12"N, 121°31'55.80"E) on 18 December 2021.

*24. *Homidiataibaiensis* Yuan & Pan, 2013

Fig. 1E

**Remarks.** New record. Collected in Shiding, New Taipei City (24°57'30.8"N, 121°39'30.2"E) on 10 October 2021, from litter of *Camelliaoleifera* (oil-seed camellia).

**25. *Lepidocyrtusheterolepis* Yosii, 1959**

**Remarks.**Yosii (1982). Locality unknown.

**26. *Lepidocyrtusscaber* Ritter, 1911**

**Remarks.**Zhao et al. (1997). Locality unknown.

**27. *Seiraoligoseta* Lee & Park, 1989**

**Remarks.** Henchun, Pintung County, from sugar cane thicket, litter of bamboo forest and poor soil under shrubs (Lee and Park 1989).

**28. *Sinellacurviseta* Brook, 1882**

Fig. 1F

**Remarks.** Cosmopolitan (Hopkin 1997). Xitou, Nantou County, from litter and soil of mixed arboreal vegetation, acorn, poor soil under shrubs and litter layer of diverse arboreal composition (Lee and Park 1989). Collected in Xiayun, Taoyuan City (24°49'40.9"N, 121°22'50.3"E) on 4 November 2020.

**29. *Sinhomidiabicolor* (Yosii, 1965)**

*Acanthocyrtusbicolor* Yosii, 1965.

*Achanturellabicolor*: Chi 1981, Lee and Park 1989.

**Remarks.** Wulai, New Taipei City and Kantaoshan, Nantou County, from litter and soil of acorn stands, on mosses and under stones (Lee and Park 1989).

**30. *Willowsiaformosana* (Denis, 1929)**

*Siraformosana* Denis, 1929.

*Seiraformosana*: Chi 1981.

**Remarks.** Taipei (Denis 1929).

**31. *Willowsiajacobsoni* (Börner, 1913)**

*Sirajacobsoni* Börner, 1913.

**Remarks.** Chung Hsing University, Taichung City, from bamboo leaf litter, arboreal vegetation, acorn stands, poor soil under shrubs, outer layer of banana trees and on mosses and under stones (Lee and Park 1989).

#### Family Isotomidae Schäffer, 1896

*32. *Folsomiacandida* Willem, 1902

Fig. 2A

**Remarks.** New record. Collected in Hanxi, Datong Township, Yilan County (24°36'35.64"N, 121°41'13.8"E) on 1 February 2021.

**Figure 2.**
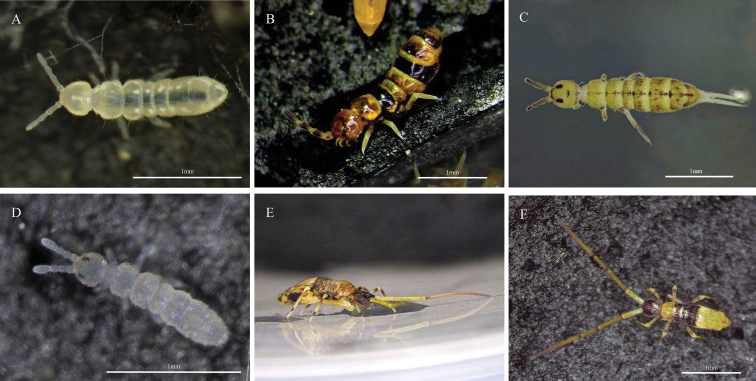
Photos of Collembola in Taiwan **A***Folsomiacandida* Willem, 1902 **B***Isotomapinnata* Börner, 1909 **C***Isotomuruspunctiferus* Yosii, 1963 **D***Proisotomaminuta* (Tullberg, 1871) **E***Callyntrurataiwanica* Yosii, 1965 (lateral view) **F***Callyntrurataiwanica* Yosii, 1965 (dorsal view).

*33. *Isotomapinnata* Börner, 1909

Fig. 2B

**Remarks.** New record. Collected in Wulai, New Taipei City (24°52'55.7"N, 121°32'10.67"E) on 30 October 2021.

**34. *Isotomatakahashii* Yosii, 1940**

*Isotomurustakahashii*: Yosii 1963.

**Remarks.** Gokwan, Xiulin Townshhip, Hualien County (Yosii 1940).

**35. *Isotomurusannectens* Yosii, 1963**

**Remarks.**Yosii (1963). Locality unknown.

*36. *Isotomuruspunctiferus* Yosii, 1963

Fig. 2C

**Remarks.** New record. Collected from rocky sea shore in Waimushan, Keelung City (25°9'48.19"N, 121°43'30.24"E) on 28 May 2022.

*37. *Proisotomaminuta* (Tullberg, 1871)

Fig. 2D

*Isotomaminuta* Tullberg, 1871.

**Remarks.** New record. Collected in Baoshan, Hsinchu County (24°44'32.73"N, 121°03'28.76"E) on 8 October 2020.

#### Family Paronellidae Börner, 1906

**38. *Callyntruraaffinis* Lee & Park, 1989**

Callyntrura (Gunungphysa) affinis Lee & Park, 1989.

**Remarks.** Reported in Manchou, Pintung County, from dry soil under shrubs (Lee and Park 1989).

**39. *Callyntrurajaponica* (Kinoshita, 1917)**

*Paronellajaponica* Kinoshita, 1917.

*Handschinphysajaponica*: Yosii 1956.

*Aphysajaponica*: Chi 1981.

**Remarks.** Zhiben Village, Beinan Township, Taitung County (Uchida 1943).

**40. *Callyntruramicrophysarum* Yosii, 1965**

*Callyntruramicrophysarum* and *Callyntruramicrophysarumstriata* Yosii, 1965.

Callyntrura (Gunungphysa) microphysarum and Callyntrura (Gunungphysa) microphysarum striata: Lee and Park 1989.

*Paronellamicrophysarum*: Chi 1981.

**Remarks.** Zhiben Village (Beinan Township, Taitung County), Meixi (Ren’ai Township, Nantou County), Chiayi County (Uchida 1943), Wulai (New Taipei City) (Yosii 1965) and Xitou (Nantou County), from litter and soil of mixed arboreal vegetation, on mosses and under stones, and from dry soil under shrubs (Lee and Park 1989).

**41. *Callyntruraspinidentata* Lee & Park, 1989**

Callyntrura (Gunungphysa) spinidentata Lee & Park, 1989.

**Remarks.** Xitou, Nantou County, from litter and soil of mixed arboreal vegetation (Lee and Park 1989).

**42. *Callyntrurataiwanica* Yosii, 1965**

Fig. 2E, F

*Paronellataiwanica*: Chi 1981.

Callyntrura (Gunungphysa) taiwanica: Lee and Park 1989.

**Remarks.** Wulai, New Taipei City, on mosses and under stones (Lee and Park 1989). Collected in Xindian, New Taipei City (24°56'47.46"N, 121°27'43.02"E) on 2 December 2021.

**43. *Cyphoderusjavanus* Börner, 1906**

*Cyphoderusassimilis*: Chi 1981.

**Remarks.** Eluanbi, Hengchun Township, Pingtung County (Uchida 1943).

**44. *Salinacelebensis* (Schäffer, 1898)**

*Cremastocephaluscelebensis* Schäffer, 1898.

**Remarks.** Manchou, Pintung County (Lee and Park 1989) and Weishang Village, Ren’ai Township, Nantou County (Yosii 1940), from dry soil under shrubs and on mosses and under stones (Lee and Park 1989).

**45. *Salina mutabilis* Lee & Park, 1989**

**Remarks.** Xitou, Nantou County, from litter and soil of mixed arboreal vegetation, soil under bamboo leaf litter and under stones (Lee and Park 1989).

#### Family Tomoceridae Schäffer, 1896

**46. *Tomoceruscuspidatus* Börner, 1909**

**Remarks.** Nenggao Village, Ren’ai Township, Nantou County and Gokwan, Xiulin Townshhip, Hualien (Yosii 1940).

**47. *Tomocerusocreatus* Denis, 1948**

Fig. 3A

**Remarks.** Locality not specified (Yosii 1977). Collected in National Taiwan University, Taipei City (25°1'12.69"N, 121°32'37.25"E) on 14 December 2021.

**Figure 3.**
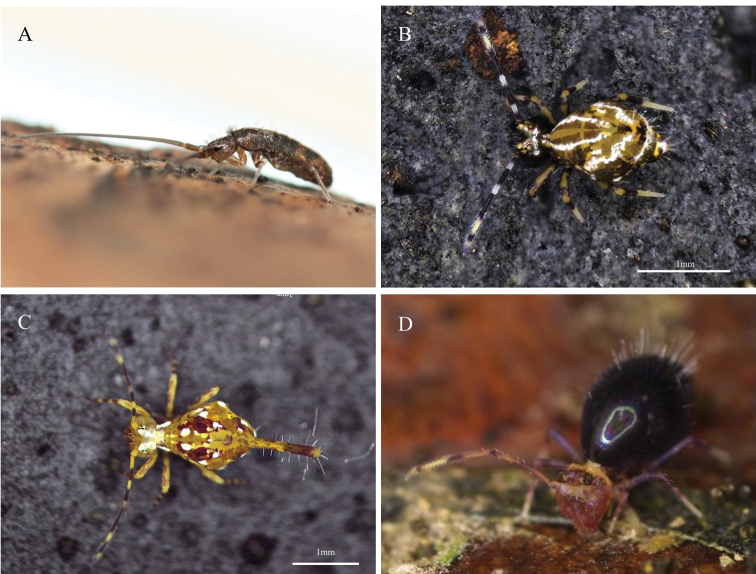
Photos of Collembola in Taiwan **A***Tomocerusocreatus* Denis, 1948 **B***Papirioidescaishijiensis* (Wu & Chen, 1996) **C***Papirioidesjacobsoni* Folsom, 1924 (spotty morph) **D***Ptenothrixcorynophora* Börner, 1909.

**Order Symphypleona Börner, 1901**

#### Family Dicyrtomidae Börner, 1906

**48. *Calvatominaformosana* (Yosii, 1965)**

*Sphyrothecaformosana* Yosii, 1965.

*Dicyrtominaformosana*: Chi 1981.

**Remarks.** Wulai, New Taipei City (Yosii 1965).

*49. *Papirioidescaishijiensis* (Wu & Chen, 1996)

Fig. 3B

Ptenothrix (Papirioides) caishijiensis Wu & Chen, 1996.

**Remarks.** New record. Collected in Lileng, Heping District, Taichung City (24°9'53.65"N, 120°57'12.62"E) on 7 November 2021.

**50. *Papirioidesmirabilis* (Denis, 1929)**

*Ptenothrixmirabilis* Denis, 1929: Chi 1981.

*Ptenothryxmirabilis*: Yosii 1940.

**Remarks.** Nanshan Village, Datong Township, Yilan County (Yosii 1940).

*51. *Papirioidesjacobsoni* Folsom, 1924

Fig. 3C

**Remarks.** New record. Specimens used for DNA barcode analysis are archived in the Collembola collection of the Museum of Zoology, National Taiwan University, Taipei, Taiwan (NTUM-COL): four specimens collected at the Huisun Experimental Forest Station, Ren’ai Township, Nantou County on 26 February 2022 (NTUM-COL-00001, 00002, 00005, 00006); one specimen collected in Neihu Dist., Taipei City on 26 December 2021 (NTUM-COL-00011); and two specimens collected in Wulai, New Taipei City on 26 December 2021 (NTUM-COL-00026, 00027). The species has two colour-morphs: a “spotty” morph with clearly separated white spots and a “milky” morph with irregular white patterns that are connected throughout the body (Fig. 5). DNA barcodes showed that the *P.jacobsoni* specimens analysed contain two genetically-distinct lineages, L1 and L2 (Fig. 5), corresponding to specimens collected in northern and central Taiwan, respectively. The mean *p*-distance between L1 and L2 is 8.3% (range: 7.6–8.8%). The “spotty” and “milky” colour-morphs can be found in both L1 and L2 and, thus, are not genetically distinct from each other. In fact, at one location, we found both the “spotty” and the “milky” morphs with identical COI sequences (NTUM-COL-00005 and 00006; Fig. 5).

*52. *Ptenothrixcorynophora* Börner, 1909

Fig. 3D

**Remarks.** New record. Collected in Houtong, New Taipei City (25°5'14.62"N, 121°49'38.95"E) on 22 November 2021.

*53. *Ptenothrixdenticulata* (Folsom, 1899)

Fig. 4A

*Papiriusdenticulatus* Folsom, 1899.

**Figure 4.**
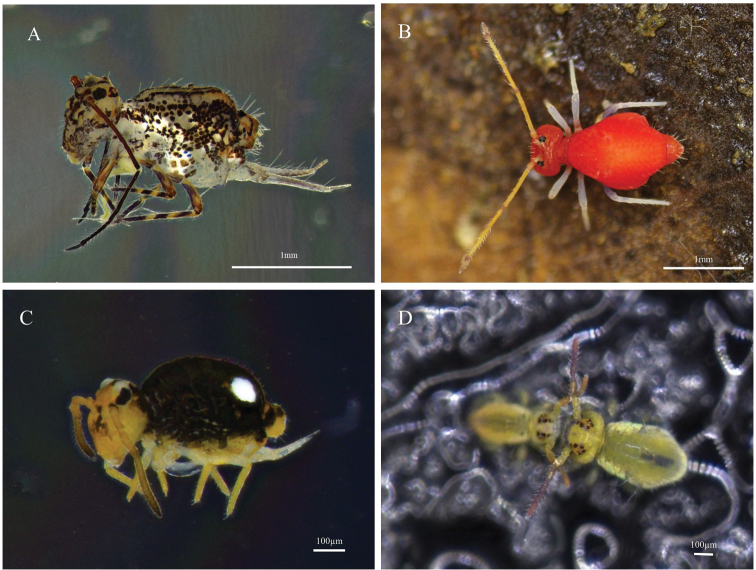
Photos of Collembola in Taiwan **A***Ptenothrixdenticulata* (Folsom, 1899) **B***Ptenothrixmonochroma* Yosii & Lee, 1963 **C***Sminthurinustrinotatus* Axelson, 1905 **D***Sminthuridespenicillifer* (Schäffer, 1896).

**Remarks.** New record. Collected in Xindian Dist., New Taipei City (24°54'53.67"N, 121°31'56.74"E) on 7 May 2022.

*54. *Ptenothrixmonochroma* Yosii & Lee, 1963

Fig. 4B

**Remarks.** New record. Collected in Sifenzi, New Taipei City (24°57'43.58"N, 121°39'46.92"E) on 28 November 2021.

#### Family Katiannidae Börner, 1913

*55. *Sminthurinustrinotatus* Axelson, 1905

Fig. 4C

**Remarks.** New Record. Collected in Chunri Township, Pingtung County (22°24'39.04"N, 120°44'16.77"E) on 5 June 2022.

#### Family Sminthuridae Lubbock, 1862

**56. *Neosminthurusamabilis* (Yosii, 1965)**

*Lipothrixamabilis* Yosii, 1965.

*Lipothrixmirabilis* (sic!) Chi 1981 lapsus.

**Remarks.** This species was collected in Taipei and described as *Lipothrixamabilis* Yosii, 1965. Although Yosii (1965) was cited in Chi (1981) when reviewing Taiwanese Collembola, this species was not included in Chi’s checklist; nor was any reason provided for the “exclusion”. Another species, *Lipothrixmirabilis* Yosii, 1965, was listed in Chi (1981), who cited Yosii (1965) as the source of the record. However, in the 1965 description of *L.mirabilis*, Yosii (1965) never mentioned anything about the presence of *L.mirabilis* in Taiwan. Thus, after carefully reviewing relevant publications, we added *N.amabilis* and removed *L.mirabilis* in the current checklist. It seems that Chi (1981) was confused by the names and listed inadvertently *mirabilis* instead of *amabilis*.

**57. *Szeptyckithecaformosana* (Yosii, 1965)**

*Sphyrothecaformosana* Yosii, 1965: Chi 1981.

**Remarks.** Wulai, New Taipei City (Yosii 1965).

#### Family Sminthurididae Börner, 1906

*58. *Sminthuridespenicillifer* (Schäffer, 1896)

*Sminthuruspenicillifer* Schäffer, 1896.

Fig. 4D

**Remarks.** New record. Collected in National Taiwan University, Taipei City (25°1'12.69"N, 121°32'37.25"E) on 11 November 2021.

**Figure 5.**
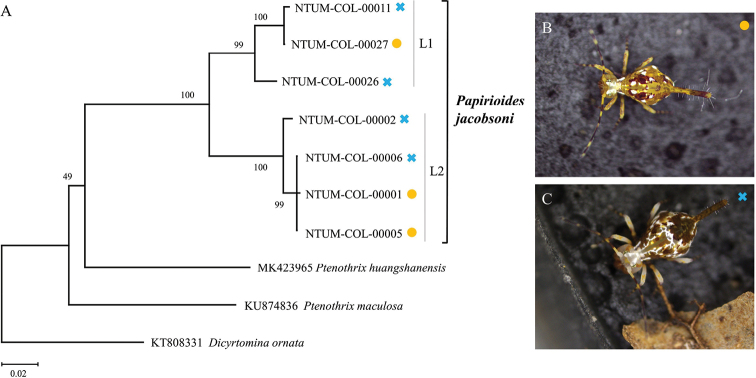
DNA barcode tree of *Papiriodesjacobsoni* in Taiwan based on neighbor-joining analysis and Kimura’s two-parameter model. The specimens analyzed form two genetically distinct lineages, L1 and L2 (**A**), corresponding to populations in northern and central Taiwan, respectively. Two color-morphs, “spotty” (**B**) and “milky” (**C**), can be found in both lineages. Specimens are labels with their NTUM catalog numbers followed by a symbol denoting their color-morphs. Numbers around nodes are bootstrap values.

## ﻿Discussion

This study is the first update of Collembola in Taiwan in more than 40 years since Chi (1981) listed 26 species in his comprehensive review. The revised checklist comprises 58 species belonging to 31 genera and 12 families, including 13 newly-recorded species, and has been used to update the Catalog of Life in Taiwan database (TaiCoL; taibnet.sinica.edu.tw). Compared to the previous checklist by Chi (1981), this list recognises four more families, including Paronellidae, Dicyrtomidae, Katiannidae and Sminthurididae and follows the most updated taxonomy for genus assignment. This comprehensive checklist serves as an overview of our most up-to-date understanding on the status of collembolan diversity and ecology in Taiwan, fills a knowledge gap resulting from the lack of taxonomic expertise for more than 40 years and provides a foundation for future collembolan studies.

Our results rejected the hypothesis that the “spotty” and “milky” colour-morphs of *Papirioidesjacobsoni* represent two distinct species and concluded that these morphological variations are intraspecific. A possible explanation for the distinct colour-morphs is sexual dimorphism. However, because the voucher specimens used for DNA extraction have become unsuitable for proper morphological examination, we are unable to test this hypothesis. In our phylogenetic results, the species consists of two genetically distinct lineages that are also geographically separated. The mean *p*-distance between the two lineages is smaller than the interspecific distances between sister species (Porco et al. 2012; Katz et al. 2015). Thus, we consider the genetic variations observed in our samples as intraspecific. Further research with additional samples is needed to understand the morphological polymorphism, genetic structure and phylogeography of this species in Taiwan.

Our field sampling was not conducted systematically. The samples we collected are mostly from the northern part of Taiwan. We also did not attempt to revisit documented locations from which the recorded species were collected in the past. Thus, we were unable to make any specific inference regarding temporal changes based on our study and previous reports. However, we can safely assume that land-use changes in the last several decades have dramatically changed the landscapes and it is likely that habitats in most documented locations have been dramatically altered. It is unclear whether any of the specimens Chi (1981) examined still exist; if they do, the specimens need to be re-examined to confirm their species identity.

The majority of the 13 species newly recorded in this study are large-bodied, atmobiotic (surface-active) species (Potapov et al. 2016), which are relatively easy to find in the field with the naked eye during a targeted search, to collect using an aspirator and to examine and store in the laboratory. Other than the 13 species, many specimens we collected and examined so far could be assigned only to a subfamily or a genus. These putative species are in the families Neanuridae, Onychiuridae, Neelidae, Tomoceridae, Isotomidae, Orchesellidae, Paronellidae, Entomobryidae, Sminthurididae, Arrhopalitidae, Sminthuridae, Bourletiellidae and Dicyrtomidae and their image records are accessible on the lead author’s Flickr page (https://flic.kr/ps/3UjMUB). Many of these presumptive species have voucher specimens archived at the NTU Museum of Zoology (preserved in 85% ethanol and stored at 4 °C). These specimens need to be further examined and barcoded to provide a more robust picture of the diversity of Collembola in Taiwan. In fact, the number of species in Taiwan, 58, is relatively low compared to those in neighbouring countries (e.g. 407 in Japan (Hishi et al. 2019)). This low number of species recorded has apparently resulted from the lack of research, as demonstrated by the 40-plus-year gap between Chi’s (1981) review and this study.

Using digital photographs for collembolan species identification, albeit unconventional, is an overlooked and under-appreciated avenue that, when used properly, can accelerate the discovery of local species diversity and improve our understanding on the global distribution of widespread species. The combination of digital photography, community science and social media platform (e.g. Collembola of Taiwan Facebook group) has become instrumental in helping us locate certain species in Taiwan and uncover morphological polymorphism in *Papirioidesjacobsoni*. We acknowledge that this approach, in general, has lower accuracy in species-level identification than conventional methods, even for large-bodied species and needs to be used with caution to avoid misidentification. Additionally, its use is likely limited to large-bodied and surface-active species, as smaller species and species living in the soil are less noticeable to the general public, harder to photograph and impossible to identify without examining detailed morphological characters (e.g. chaetotaxy) under a microscope.

## ﻿Conclusions

Fifty-eight species of Collembola belonging to 31 genera and 12 families have been reported in Taiwan, including 13 species newly recorded in this study. These numbers mark a 123% increase in species richness from the previous comprehensive review. The results of this study have been used to update the “Catalog of Life in Taiwan” (taibnet.sinica.edu.tw) and the species information in the “Checklist of the Collembola of the World” (collembola.org). Additionally, although the dicyrtomid species *Papirioidesjacobsoni* was shown to comprise two divergent mitochondrial lineages, these lineages are not concordant with morphological differences in colour morphs. Finally, we highlighted the potential and limitation of using macro photographs to reach species-level identification in Collembola.

## References

[B1] Aoki JI (2015) Pictorial keys to soil animals of Japan. Tokai University Press 2: 1386–1387.

[B2] Asahina S Yasumatsu K Ishihara T (1965) Iconographia Insectorum Japonicorum Colore Naturali Edita. Vol. 3. Hokuryukan, Tokyo, 358 pp. [In Japanese]

[B3] Axelson WM (1905) Einige neue Collembolen aus Finnland. Zoologischer Anzeiger 28: 788–794.

[B4] Bellinger PF Christiansen KA Janssens F (1996–2022) Checklist of the Collembola of the world. http://www.collembola.org

[B5] Bonet F (1930) Sur quelques Collemboles de l’Inde. Junta para la Ampliación de Estudios e Investigaciones Científicas 6(3): 249–273.

[B6] Börner C (1901) Voläufige Mitteilung über einige neue Aphorurinen und zur Systematik der Collembola. Zoologischer Anzeiger 24(633): 1–15.

[B7] Börner C (1906) Das System der Collembolen nebst Beschreibung neuer Collembolen des Hamburger Naturhistorischen Museums. Mitteilungen aus den Naturhistorischen Museum in Hamburg 23: 147–188.

[B8] Börner C (1909) Japans Collembolenfauna. (Vorläufige Mitteilung). Sitzungsberichte der Gesellschaft Naturforschender Freunde zu Berlin 2: 99–135.

[B9] Börner C (1913) Die Familien der Collembolen. Zoologischer Anzeiger 41: 315–322.

[B10] Bretfeld G (1999) Symphypleona. In: Dunger W (Ed.) Synopses on Palaearctic Collembola, vol. 2.Abhandlungen und Berichte des Naturkundemuseum Görlitz 71: 1–318.

[B11] Brook G (1882) On a new genus of Collembola (*Sinella*) allied to *Degeeria*, Nicolet. Zoological Journal of the Linnean Society 16(95): 541–545. 10.1111/j.1096-3642.1882.tb02398.x

[B12] Chen JX Christiansen K (1996) A new species of *Ptenothrix* from China (Collembola: Dicyrtomidae). The Florida Entomologist 79(4): 586–591. 10.2307/3496072

[B13] Chen YF Chung FY Hsu PC Lin CH Chen YC (2020) A preliminary study on soil arthropods of different forest types in Xitou, Taiwan. Journal of the Experimental Forest of National Taiwan University 34(3): 213–226. 10.6542/2fEFNTU.202009_34(3).0003

[B14] Chi H (1981) Literature review of Collembola in Taiwan. Annual of Taiwan Museum 24: 105–112.

[B15] Denis J (1929) Collemboles d’Extrême-Orient. Notes sur les Collemboles récoltés dans ses voyages par le Prof. F. Silvestri (I). Bolletino del Laboratorio di Zoologia Portici 22: 166–171.

[B16] Denis JMR (1934) Collemboles d’Indochine récoltés par CN Dawydoff (1^re^ note préliminaire) [Achorutini]. Bulletin de la Société Entomologique de France 39(8): 117–122. 10.3406/bsef.1934.14714

[B17] Denis J (1948) Collemboles d’Indochine. Notes d’Entomologie Chinoise 12: 183–311.

[B18] Folmer O Black M Hoeh W Lutz R Vrijenhoek R (1994) DNA primers for amplification of mitochondrial cytochrome *c* oxidase subunit I from diverse metazoan invertebrates. Molecular Marine Biology and Biotechnology 3: 294–299. 7881515

[B19] Folsom JW (1898) Japanese Collembola (Part I). The Bulletin of the Essex Institute 24: 51–57. 10.5962/bhl.part.14789

[B20] Folsom JW (1899) Japanese Collembola (Part II). Proceedings of the American Academy of Arts and Sciences 34(9): 261–274. 10.2307/20020884

[B21] Folsom JW (1924) East Indian Collembola. Bulletin of the Museum of Comparative Zoology 65: 505–517.

[B22] Gervais MP (1842) Communique une quinzaine d’espèces. Annales de la Société Entomologique de France 11: 45–49. https://gallica.bnf.fr/ark:/12148/bpt6k63364141/

[B23] Gisin H (1950) Notes sur les Collemboles avec une espèce, un nom et trois synonymes nouveaux. Mitteilungen der Schweizerische Entomologische Gesellschaft 23(4): 411–416.

[B24] Handschin E (1929) Beiträge zur Collembolenfauna von Süd-Indien. Revue Suisse de Zoologie 36: 229–262. 10.5962/bhl.part.117939

[B25] Hishi T Fujii S Saitoh S Yoshida T Hasegawa M (2019) Taxonomy, distribution and trait data sets of Japanese Collembola. Ecological Research 34(4): 444–445. 10.1111/1440-1703.12022

[B26] Hopkin SP (1997) Biology of the springtails (Insecta: Collembola). Oxford University Press, Oxford, 330 pp.

[B27] Huang C-Y Wu W-Y Chang C-P Tsao S Yuan PB Lin C-W Xia KY (1997) Tectonic evolution of accretionary prism in the arc-continent collision terrane of Taiwan. Tectonophysics 281(1–2): 31–51. 10.1016/S0040-1951(97)00157-1

[B28] Huang C-Y Yuan PB Lin C-W Wang TK Chang C-P (2000) Geodynamic processes of Taiwan arc-continent collision and comparison with analogs in Timor, Papua New Guinea, Urals and Corsica. Tectonophysics 325(1–2): 1–21. 10.1016/S0040-1951(00)00128-1

[B29] Jordana R (2012) Capbryinae and Entomobryini. In: Dunger W Bukhardt U (Eds) Synopses on Palaearctic Collembola, Volume 7/1.Soil Organisms 84(1), 1–390.

[B30] Katz AD Giordano R Soto-Adames FN (2015) Operational criteria for cryptic species delimitation when evidence is limited, as exemplified by North American Entomobrya (Collembola: Entomobryidae). Zoological Journal of the Linnean Society 173(4): 818–840. 10.1111/zoj.12220

[B31] Kimura M (1980) A simple method for estimating evolutionary rates of base substitutions through comparative studies of nucleotide sequences. Journal of Molecular Evolution 16(2): 111–120. 10.1007/BF01731581 7463489

[B32] Kinoshita S (1917) Two new species of Collembola from Japan. Zoologocal Magazine Tokyo 29: 40.

[B33] Kumar S Stecher G Li M Knyaz C Tamura K (2018) MEGA X: Molecular Evolutionary Genetics Analysis across computing platforms. Molecular Biology and Evolution 35(6): 1547–1549. 10.1093/molbev/msy096 29722887 PMC5967553

[B34] Larkin MA Blackshields G Brown NP Chenna R McGettigan PA McWilliam H Valentin F Wallace IM Wilm A Lopez R Thompson JD Gibson TJ Higgins DG (2007) Clustal W and Clustal X version 2.0. Bioinformatics 23(21): 2947–2948. 10.1093/bioinformatics/btm404 17846036

[B35] Latreille (1804) Histoire naturelle, générale et particulière des crustacés et des insectes. Vol.3. F. Dufart, Paris, 467 pp.

[B36] Lee BH Kim JT (1990) Systematic studies on Chinese Collembola (Insecta): II. Five new species and two new records from Taiwan in the family Neanuridae. Animal Systematics, Evolution and Diversity 6(2): 235–249. https://www.koreascience.or.kr/article/JAKO199011920826894.page?&lang=en

[B37] Lee BH Park KH (1989) Systematic studies on Chinese Collembola (Insecta), I. Four new species and three new records of Entomobryidae from Taiwan. Formosan Entomologist 9: 263–282. 10.6660/2fTESFE.1989025

[B38] Linnaeus C (1758) Systema natura. Laurentii Salvii, Holmiae, 824 pp.

[B39] Lubbock J (1862) Notes on the Thysanura. – Part I. Smynthuridæ. Transactions of the Linnean Society of London 23(3): 429–448. 10.1111/j.1096-3642.1860.tb00141.x

[B40] Lubbock J (1867) Notes on the Thysanura Part III. Transactions of the Linnean Society 26(1): 295–304. 10.1111/j.1096-3642.1968.tb00508.x

[B41] Lubbock J (1870) Notes on the Thysanura. – Part IV. Transactions of the Linnean Society of London 27(2): 277–297. 10.1111/j.1096-3642.1870.tb00214.x

[B42] Nicolet H (1842) Recherches pour Servir á l’Histoire des Podurelles. Nouveaux Mémoires de la Société Helvétique des Sciences Naturelles 6: 1–88.

[B43] Ono Y Aoki T Hasegawa H Dali L (2005) Mountain glaciation in Japan and Taiwan at the global Last Glacial Maximum. Quaternary International 138: 79–92. 10.1016/j.quaint.2005.02.007

[B44] Orgiazzi A Bardgett RD Barrios E (2016) Global Soil Biodiversity Atlas. European Commission, Luxembourg, 176 pp. 10.1093/oso/9780199668564.003.0007

[B45] Oudemans JT (1890) Apterygota des Indischen Archipels. Zoologische Ergebnisse 1: 73–91.

[B46] Porco D Potapov M Bedos A Busmachiu G Weiner WM Hamra-Kroua S Deharveng L (2012) Cryptic diversity in the ubiquist species *Parisotomanotabilis* (Collembola, Isotomidae): A long-used chimeric species? PLoS ONE 7(9): e46056. 10.1371/journal.pone.0046056 PMC3458803 23049931

[B47] Potapov M (2001) Isotomidae. In: Dunger W (Ed.) Synopses on Palaearctic Collembola: Vol, 3.Abhandlungen und Berichte des Naturkundemuseums Gorlitz 73: 1–603.

[B48] Potapov AA Semenina EE Korotkevich AY Kuznetsova NA Tiunov V (2016) Connecting taxonomy and ecology: Trophic niches of collembolans as related to taxonomic identity and life forms. Soil Biology & Biochemistry 10: 20–31. 10.1016/j.soilbio.2016.07.002

[B49] Potapov A Bellini B Chown S Deharveng L Janssens F Kováč Ľ Kuznetsova N Ponge JF Potapov M Querner P (2020) Towards a global synthesis of Collembola knowledge: Challenges and potential solutions. Soil Organisms 92(3): 161–188.

[B50] Ritter W (1911) Neue Thysanuren und Collembolen aus Ceylon and Bombay, gesammelt von Dr Uzel. Annalen des Naturhistorischen Museums in Wien 24: 379–398.

[B51] Salmon JT (1964) An index to the Collembola. Royal Society of New Zealand Bulletin 7: 145–644.

[B52] Schäffer C (1896) Die Collembolen der Umgebung von Hamburg und benachbarter Gebiete. Mitteilungen aus dem Naturhistorishen Museum in Hamburg 13: 149–216.

[B53] Schäffer C (1898) Die Collembola des Bismarck-Archipels nach der Ausbeute von Prof. Dr. F. Dahl. Archiv für Naturgeschichte 64: 393–425.

[B54] Schött H (1891) Beiträge zur Kenntniss Kalifornischer Collembola. Bihang Till Kungliga Svenska vetenskapsakademiens Handlingar, 26 pp.

[B55] Shi SD Pan ZX Qi X (2009) A new species of the genus *Homidia* Börner, 1906 (Collembola: Entomobryidae) from East China. Zootaxa 2020(1): 63–68. 10.11646/zootaxa.2020.1.4

[B56] Shi SD Pan ZX Zhang F (2010) A new species and a new record of the genus *Homidia* Börner, 1906 from East China (Collembola: Entomobryidae). Zootaxa 2351(1): 29–38. 10.11646/zootaxa.2351.1.3

[B57] Shiraki T (1932) Collembola. In: Pictorial handbook on Japanese insects. Hokuryukan, Tokyo, 2115–2126. [In Japanese]

[B58] Shiraki T (1954) Collembola. In: Taxonomy of insects. Hokuryukan, Tokyo, 7–21. [In Japanese]

[B59] Takano S Yanagihara M (1939) Pests and beneficial insects of sugar cane in Taiwan. Taiwan sugar cane society, 294 pp.

[B60] Tullberg T (1871) Forteckning ofver Svenska Podurider. Öfvers K Vetens Akad Förh 28: 143–152.

[B61] Uchida H (1943) On some Collembola-arthropleona from Nippon. Bulletin of the National Science Museum 8: 1–18.

[B62] Uchida H (1955) Synopsis of the Apterygota of Japan and its vicinity. Historical reviews of the study on the Apterygota of the Far East and remarks on the geographical distribution of the Far Eastern Collembola. Nihon Seibutsu Chiri Gakkai Kaiho 16–19: 197–203.

[B63] Uchida H (1956) Synopsis of the Apterygota of Japan and its vicinity. (III). Ordo Collembola, genus *Podura* to genus *Anurida*. Papers in Science Reports. Hirosaki University 3(1): 25–29.

[B64] Uchida H (1957a) Synopsis of the Apterygota of Japan and its vicinity. (IV). Ordo Collembola, genus *Lobella* to genus *Ballistura*. Papers in Science Reports. Hirosaki University 4(1): 18–25.

[B65] Uchida H (1957b) Synopsis of the Apterygota of Japan and its vicinity. (V). ordo Collembola, genus *Ballistura* to genus *Homidia*. Papers in Science Reports. Hirosaki University 4(2): 38–45.

[B66] Uchida H (1958a) Synopsis of the Apterygota of Japan and its vicinity. (VI). Ordo Collembola, genus *Sira* to genus *Cyphoderus*. Papers in Science Reports. Hirosaki University 5(1): 13–20.

[B67] Uchida H (1958b) Synopsis of the Apterygota of Japan and its vicinity. (VII). Ordo Collembola, genus *Megalothorax* to genus *Deuterosminthurus*. Papers in Science Reports. Hirosaki University 5(2): 33–35.

[B68] Uchida H (1959a) Synopsis of the Apterygota of Japan and its vicinity. (IX). Bibliography. 1. Papers in Science Reports. Hirosaki University 6(2): 44–50.

[B69] Uchida H (1959b) Synopsis of the Apterygota of Japan and its vicinity. (VIII). Ordo Collembola, genus *Sminthurus* to genus *Ptenothrix*. Papers in Science Reports. Hirosaki University 6(1): 22–26.

[B70] Uchida H (1960) Synopsis of the Apterygota of Japan and its vicinity. (X). Bibliography. 2. Papers in Science Reports. Hirosaki University 7(1): 10–16.

[B71] Usinger RL (1956) Aquatic insects of California: with keys to North American genera and California species. University of California Press, 507 pp. 10.5962/bhl.title.61952

[B72] Willem V (1902) Note préliminaire sur les Collemboles des Grottes de Han et de Rochefort. Annales de la Société Entomologique de Belgique 46: 275–283.

[B73] Wu M Chen J (1996) A new species of the subgenus Papirioides from China (Collembola: Dicyrtomidae). Insect Science 3(2): 138–144. 10.1111/j.1744-7917.1996.tb00219.x

[B74] Yoshii R (1995) Identity of some Japanese Collembola III. Acta Zoologica Asiae Orientalis 3: 51–68.

[B75] Yosii R (1940) On some Collembola from Formosa. Annotationes Zoologicae Japonenses 19(2): 114–118. https://ci.nii.ac.jp/naid/110003352432

[B76] Yosii R (1954) Höhlencollembolen Japans I. Kontyû 20(3–4): 62–70.

[B77] Yosii R (1956) Monographie zur Höhlencollembolen Japans. Contributions from the Biological Laboratory, Kyoto University 3: 1–109.

[B78] Yosii R (1959) Studies on the collembolan fauna of Malay and Singapore with special reference to the Genera: *Lobella*, *Lepidocyrtus* and *Callyntrura*. Contributions from the Biological Laboratory Kyoto University 10: 1–65. https://ci.nii.ac.jp/naid/110003352432

[B79] Yosii R (1963) On some Collembola of Hindukush, with notes on *Isotoma* Bourlet and its allies. Results of the Kyoto University Scientific Expedition to the Karakoram and Hindukush 1955(4): 3–42.

[B80] Yosii R (1965) On some Collembola of Japan and adjacent countries. Contributions from the Biological Laboratory, Kyoto University 19: 1–71. http://hdl.handle.net/2433/155937

[B81] Yosii R (1966) On some Collembola of Afghanistan, India and Ceylon, collected by the Kuphe-Expedition, 1960. Results of the Kyoto University Scientific Expedition to the Karakoram and Hindukush 3: 333–405.

[B82] Yosii R (1976) On some Neanurid Collembola of Southeast Asia. Nature and Life in Southeast Asia. Japan Society for the Promotion of Science 7: 291–298.

[B83] Yosii R (1977) Critical check list of the Japanese species of Collembola. Contributions from the Biological Laboratory, Kyoto University 25(2): 141–170. http://hdl.handle.net/2433/156007

[B84] Yosii R (1982) Lepidocyrtid Collembola of Sabah. Entomological Report from the Sabah Forest Research Center 5: 1–47.

[B85] Yosii R Lee CE (1963) On some Collembola of Korea, with notes on the genus *Ptenothrix*. Contributions from the Biological Laboratory Kyoto University 15: 1–37.

[B86] Yuan X Pan ZX (2013) Two new species of Entomobryidae (Collembola) of Taibai Mountain from China. ZooKeys 338: 67–81. 10.3897/zookeys.338.5723 PMC3800841 24146584

[B87] Zhao L Tamura H Ke X (1997) Tentative checklist of Collembolan species from China (Insecta). Publications of Itako Hydrobiological Station 9: 15–40. https://jglobal.jst.go.jp/en/detail?JGLOBAL_ID=200902180307909383

